# Clinical validation of commercial deep-learning based auto-segmentation models for organs at risk in the head and neck region: a single institution study

**DOI:** 10.3389/fonc.2024.1375096

**Published:** 2024-07-11

**Authors:** Casey L. Johnson, Robert H. Press, Charles B. Simone, Brian Shen, Pingfang Tsai, Lei Hu, Francis Yu, Chavanon Apinorasethkul, Christopher Ackerman, Huifang Zhai, Haibo Lin, Sheng Huang

**Affiliations:** ^1^ New York Proton Center, New York, NY, United States; ^2^ National Clinical Research Center for Cancer, Tianjin’s Clinical Research Center for Cancer, Tianjin Medical University Cancer Institute & Hospital, Tianjin, China

**Keywords:** deep-learning, autosegmentation, head&neck cancer, OARs, radiotherapy

## Abstract

**Purpose:**

To evaluate organ at risk (OAR) auto-segmentation in the head and neck region of computed tomography images using two different commercially available deep-learning-based auto-segmentation (DLAS) tools in a single institutional clinical applications.

**Methods:**

Twenty-two OARs were manually contoured by clinicians according to published guidelines on planning computed tomography (pCT) images for 40 clinical head and neck cancer (HNC) cases. Automatic contours were generated for each patient using two deep-learning-based auto-segmentation models—Manteia AccuContour and MIM ProtégéAI. The accuracy and integrity of autocontours (ACs) were then compared to expert contours (ECs) using the Sørensen-Dice similarity coefficient (DSC) and Mean Distance (MD) metrics.

**Results:**

ACs were generated for 22 OARs using AccuContour and 17 OARs using ProtégéAI with average contour generation time of 1 min/patient and 5 min/patient respectively. EC and AC agreement was highest for the mandible (DSC 0.90 ± 0.16) and (DSC 0.91 ± 0.03), and lowest for the chiasm (DSC 0.28 ± 0.14) and (DSC 0.30 ± 0.14) for AccuContour and ProtégéAI respectively. Using AccuContour, the average MD was<1mm for 10 of the 22 OARs contoured, 1-2mm for 6 OARs, and 2-3mm for 6 OARs. For ProtégéAI, the average mean distance was<1mm for 8 out of 17 OARs, 1-2mm for 6 OARs, and 2-3mm for 3 OARs.

**Conclusions:**

Both DLAS programs were proven to be valuable tools to significantly reduce the time required to generate large amounts of OAR contours in the head and neck region, even though manual editing of ACs is likely needed prior to implementation into treatment planning. The DSCs and MDs achieved were similar to those reported in other studies that evaluated various other DLAS solutions. Still, small volume structures with nonideal contrast in CT images, such as nerves, are very challenging and will require additional solutions to achieve sufficient results.

## 1 Introduction

The evolution of radiation therapy techniques in recent decades has led to major improvements in dose conformality along with precision in dose delivery. Modern methods of dose delivery such as intensity-modulated radiation therapy, volumetric arc therapy, and recently, intensity-modulated proton therapy all have proven to improve local control as well as normal tissue sparing in various tumor types (1–4).

However, to capitalize on the benefits of these treatment modalities, target volumes and surrounding organs-at-risk (OARs) must be carefully delineated on computed tomography (CT) images. This is heavily demonstrated in the case of head and neck cancer (HNC) that often lie in complex anatomical locations surrounded by numerous OARs. The delineation of neighboring structures is a time-consuming manual process, that mandates experienced knowledge of the local anatomy. Furthermore, manual delineation introduces inter-observer variability as evidenced in several recent studies (5–7). OAR delineation guidelines have been published by many authors to combat this but vary widely causing difficulty when comparing dose-volume relationships across studies (8). Consensus guidelines were established in 2015 integrating advice and expertise from radiation oncologists from across the world (9). However, even with established evidence-based guidelines, inter-observer variability still exists as shown in a study conducted by van der Veen et al. (10)in which only around half of the participating radiation oncologists utilized the standardized guidelines.

Methods of utilizing advancements in automatic segmentation techniques have emerged to potentially combat lengthy processing times and wide inter-observer variability. Atlas-based auto-segmentation (ABAS) is one such method in which an ‘atlas’ of OARs is established by training a software program with a dataset that has OARs already labeled. An explanation of this process has been published by Han et al. (11). This technique has been proven to reduce processing time as well as generating appropriate sedimentation for various OARs (12, 13). Another automated segmentation method currently being investigated is deep-learning-based auto-segmentation (DLAS). DLAS utilizes machine learning to incorporate vast datasets and generate an automated solution. This technique has shown promise in recent studies assessing the efficiency in the head and neck region (14, 15).

There are several commercially available ABAS software programs as well as in-house developed DLAS programs that have been validated on HNC. A comparison study conducted by La Macchia et al. (16) compared three auto-segmentation programs and reported significant reductions in time to generate quality contours when compared to manual processes. However, many studies have shown the contours generated by either ABAS or DLAS methods still require additional manual editing to be clinically acceptable (16–18). In a study conducted by van Dijk et al. (19), time to generate clinically acceptable contours still proved to be significantly less when created using either ABAS or DLAS. Even still, the authors noted that the DLAS method used outperformed ABAS when evaluating on a cohort of HNC patients. However, large cohorts of training data sets are required to train and get an accurate DLAS model. It is not feasible for each center to develop and train their own DLAS model, thus commercially available models or shared DLAS packages would be advantageous. Evidence from clinical validation of DLAS packages will allow for centers to identify solutions that will provide optimal performance for their particular needs. Thus, this study aims to clinically validate the generic models for HNC OAR autocontouring of two commercially available deep-learning-based auto-segmentation software packages, AccuContour (version 3.1, Manteia Medical Technologies, Wisconsin, MI) and ProtègèAI (version 1.0, MIM Software Inc., Cleveland, OH), Both MIM and Manteia’s solutions include a generic HNC autocontouring model. Quantitative evaluation on a set of 40 clinical HNC patients will be performed.

## 2 Materials and methods

### 2.1 Expert contour creation

To validate each DLAS model, a cohort of 40 HNC patients were selected who previously received treatment at our institution. All patient data in this retrospective study was approved under an internal review board. All planning CT (pCT) images consisted of 512 pixels × 512 pixels in each slice with voxel size of 0.98mm×0.98mm×1.50mm. All the CT data were acquired on the same version of CT scanner (Somatom Definition AS, Siemens, Forchheim, Germany) without contrast enhancement. In total, 22 OARs were manually contoured to establish the expert contours (ECs) on the pCT images by expert radiation oncologists at our institution according to international consensus guidelines. These OARs were divided into five groups: 1) *Glandular*: submandibular and parotid glands; 2) *Aerodigestive Tract*: oral cavity, larynx, esophagus, constrictor muscle; 3) *Ocular and Aural*: cochlea, lens, eye; 4) *Neural*: brainstem, chiasm, spinal cord, optic nerves, temporal lobes; 5) *Other*: mandible.

### 2.2 DLAS contour creation

In contrast to atlas model-based auto-segmentation which utilizes a trained model of shape and appearance characteristics of anatomy structures and then project onto a new image set through deformable imaging registration, DLAS uses deep neural network architectures with multiple (2 or more) hidden layers to learn features from a dataset by modeling complex nonlinear relationships. These architectures are usually formed by stacking several different-type layers that transform input images to the desired output. The transformation through convolution filters, or kernels, reveals local connectivity between neurons of adjacent layers exploiting spatially local correlation. This permits the networks to learn features both globally and locally allowing the network to detect subtle variations in the input data, which here mean the features of different OARs. The training processes generally utilize supervised learning by back-propagation algorithms, which optimize the node weights to minimize the loss between the predicted and known output through each training iteration to a satisfactory level of accuracy. It remains to be unanswered how many patient scans are optimal to produce clinically acceptable results. One would agree that a robust dataset that includes a large variability of patient anatomies would achieve reasonable and robust model. During past years, more and more commercial DLAS software have emerged and become clinically available. The following two different DLAS packages were implemented in our clinic and assessed in this study:

Manteia AccuContour is a commercial deep-learning-based auto-segmentation software using deep convolutional neural network models based on a U-Net architecture, the design of which follows the work of Ronneberg et al. (20). The training data included in the model consists of 100 HNC image sets acquired from GE, Philips, and Siemens CT scanners. The HNC model was then applied to the same 40 pCTs used in the expert contour creation. As with the expert contour creation, a unique set of autocontours (ACs) was generated for each patient case.

The MIM ProtégéAI generic HNC model is a cloud-based deep learning segmentation model with a similar structure to U-Net. The training data included in the model consists of about 400 HNC images gathered from 31 institutions mainly across the US, but with a few additional institutions located in Europe, Hong Kong, and Australia. MIM’s HNC model was also applied to the 40 pCTs used in the expert contour creation. No post-processing was completed after the 3D volume generation for each contour. Again, a unique set of ACs was generated for each patient case. The MIM ProtégéAI autocontour model did not provide contours for temporal lobes, cochlea, or the constrictor muscle. Thus, only 17 OAR contours were generated for each patient.

### 2.3 Evaluation metrics

For performance evaluation, the Sørensen-Dice similarity coefficient (DSC) (21), and the mean distance (MD) between the ECs and ACs for the OARs of each patient were calculated as a comparison metric. The DSC is defined as:


(1) $$DSC\left(A,B\right)=\;\frac{2\left|A\cap^{}B\right|}{\left|\left|A\right|+\left|B\right|\right|}$$

Which describes the overlapping volume between two structures *A* and *B*. A value of 0 indicates no overlap; a value of 1 indicates complete overlap. The MD is a bi-directional measure of the distance between the surface of two contours and is defined as:


(2) $$MD\left(A,\;B\right)\;=\;\frac{1}{N\left(A\right)}\sum_{i=1}^{N\left(A\right)}\min d\left(a_{i},b\in S_{B}\right)\;$$

Where *N(A)* is the total nodes on the surface of *A* structure, min *d(a*, $b\in S_{B}$ *)* is the minimum Euclidean distance of node $a_{i}\;$ to any point b on surface of *B*. The smaller mean distance indicates the surfaces of *A* and *B* are closer to each other. For every clinical case, each OAR delineated on the ECs and ACs was compared, generating a DSC and MD value. Results are presented as averages across the patient cohort for each OAR with ranges describing the performance of grouped OARs and mean ± standard deviation describing the performance of specific OARs.

## 3 Results

### 3.1 Manteia AccuContour evaluation

The time to autocontour all 22 OARs using Manteia AccuContour was 1min/patient. **Table 1** lists the average DSC and MD values between ECs and Manteia AccuContour ACs for each OAR.

**Table 1:** DSCs and MDs for multiple-subject Manteia AccuContour ACs vs. ECs.

Variable*	OAR					
	Brainstem	Chiasm	Cochlea L	Cochlea R	Constrictor Muscle	Esophagus
**DSC**	0.84 ± 0.05	0.28 ± 0.14	0.68 ± 0.13	0.68 ± 0.10	0.55 ± 0.08	0.75 ± 0.11
**MD (mm)**	0.88 ± 0.32	2.20 ± 1.28	0.24 ± 0.14	0.26 ± 0.12	1.98 ± 2.81	1.79 ± 6.74
	Eye L	Eye R	Larynx	Lens L	Lens R	Mandible
**DSC**	0.83 ± 0.05	0.83 ± 0.05	0.42 ± 0.10	0.68 ± 0.11	0.70 ± 0.10	0.90 ± 0.07
**MD (mm)**	0.70 ± 0.26	0.67 ± 0.27	2.65 ± 0.79	0.19 ± 0.15	0.16 ± 0.11	0.43 ± 0.15
	Optic Nerve L	Optic Nerve R	Oral Cavity	Parotid L	Parotid R	Spinal Cord
**DSC**	0.64 ± 0.08	0.62 ± 0.10	0.80 ± 0.09	0.78 ± 0.10	0.77 ± 0.11	0.78 ± 0.06
**MD (mm)**	0.47 ± 0.30	0.96 ± 0.61	2.31 ± 0.91	1.75 ± 1.13	1.88 ± 1.15	1.80 ± 5.43
	Submandibular L	Submandibular R	Temporal Lobe L	Temporal Lobe R		
**DSC**	0.73 ± 0.19	0.73 ± 0.17	0.78 ± 0.09	0.78 ± 0.09		
**MD (mm)**	2.05 ± 5.67	1.72 ± 3.63	2.78 ± 1.95	2.80 ± 2.02		

*Mean ± Standard Deviation.

**Figure 1:**
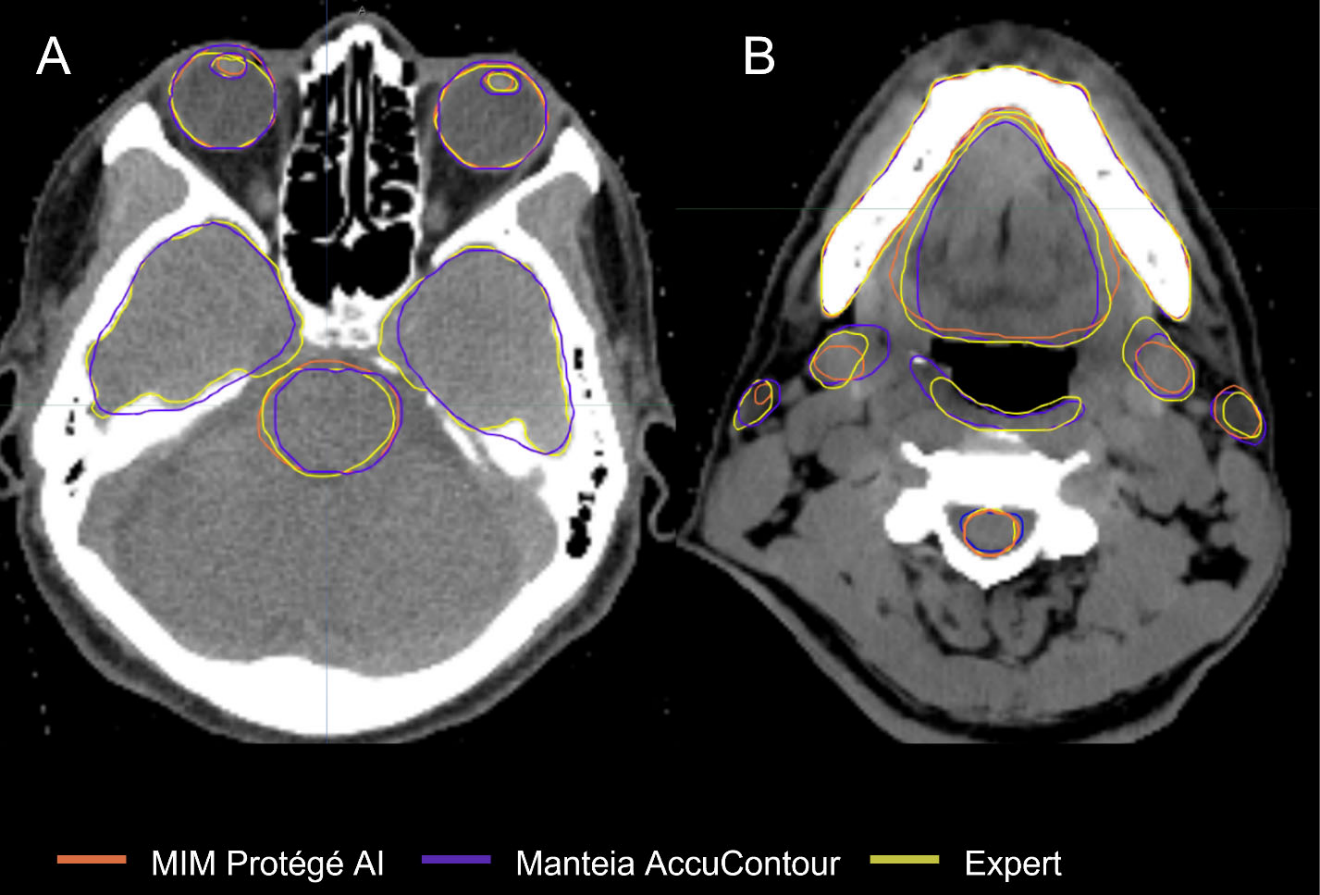
Comparison of ACs and ECs for, **(A)** Brainstem, Temporal Lobes, Eyes, Lenses; **(B)** Spinal Cord, Constrictor Muscle, Submandibular Glands, Parotid Glands, Oral Cavity, Mandible.

○ *Glandular* OARs: the AccuContour model showed high DSCs and contour agreement (0.73-0.78). The MDs were similarly acceptable for both left and right parotid glands (1.75 ± 1.13, 1.88 ± 1.15mm, respectively). The left submandibular gland MDs were slightly larger than the right submandibular gland (2.05 ± 5.67, 1.72 ± 3.63mm, respectively).
○ *Aerodigestive Tract* OARs: the AccuContour model generated the best DSCs for the oral cavity (see **Figure 1B**) and esophagus (0.80 ± 0.09, 0.75 ± 0.11, respectively), while generating lower DSCs for the constrictor muscle and larynx (0.55 ± 0.08, 0.42 ± 0.10, respectively). The MDs for these OARs were relatively large (1.79-2.65mm).
○ *Ocular and Aural* OARs: AccuContour ACs resulted in high DSCs for both eyes (0.83 ± 0.05) (see **Figure 1A**), and moderate DSCs for the left and right lens and cochlea (0.68-0.70). The MDs for all these OARs were low (0.16-0.70mm).
○ *Neural* OARs: the AccuContour model showed high DSCs for the brainstem and spinal cord (0.84 ± 0.05, 0.78 ± 0.06, respectively). Both the right and left optic nerve were lower (0.62 ± 0.10, 0.64 ± 0.08, respectively), and the chiasm performed the lowest (0.28 ± 0.14) (see **Figure 1A**). The resulting MDs were low for the brainstem and right and left optic nerve (0.47-0.88mm) but were higher for the spinal cord and chiasm (1.80-2.20mm).
○ *Mandible* OAR: ACs generated by AccuContour resulted in high DSCs (0.90 ± 0.07) and a similarly acceptable MD (0.43 ± 0.15mm).

### 3.2 MIM ProtègèAI evaluation

The average time to autocontour all 17 OARs using MIM ProtègèAI was 5min/patient. **Table 2** lists the average DSC and MD values between ECs and MIM ProtègèAI ACs for each OAR.

o *Glandular* OARs: the ProtégéAI model showed high DSCs for both left and right parotid glands (0.77 ± 0.13, 0.79 ± 0.09, respectively) (**Figure 1B**). The MDs were similarly acceptable for both the left and right parotid glands (1.31 ± 0.74, 1.44 ± 1.57mm, respectively).
o *Aerodigestive Tract* OARs: the ProtégéAI model produced the best DSCs for the oral cavity (**Figure 1B**) and esophagus (0.82 ± 0.06, 0.75 ± 0.11, respectively), but resulted in lower DSCs for the larynx (0.65 ± 0.09). The MDs for the esophagus were relatively low (0.76 ± 0.50mm) but were higher for the oral cavity and larynx (2.04 ± 0.71, 2.51 ± 0.97mm respectively).
o *Ocular and Aural* OARs: ProtégéAI ACs resulted in high DSCs for both right and left eyes (0.89 ± 0.02, 0.89 ± 0.03, respectively) (**Figure 1A**), but performed less effectively for the left and right lenses (0.55 ± 0.27, 0.54 ± 0.29, respectively). However, the MDs for these OARs were low (0.43-0.62mm).
o *Neural* OARs: the ProtégéAI model showed high DSCs for the brainstem and spinal cord (0.81 ± 0.04, 0.79 ± 0.05, respectively). Like AccuContour, the right and left optic nerve DSCs were lower (0.63 ± 0.13, 0.53 ± 0.09, respectively), and again, the chiasm performed the lowest (0.30 ± 0.14) (**Figure 1A**). The resulting MDs were low for the left and right optic nerves (0.54 ± 0.34, 0.40 ± 0.22mm, respectively), but were slightly higher for the brainstem, chiasm, and spinal cord (1.04-1.49mm).
o *Mandible* OAR: ACs generated by ProtégéAI resulted in high DSCs (0.91 ± 0.03) and similarly acceptable MDs (0.62 ± 0.32mm).

**Table 2:** DSCs and MDs for multiple-subject MIM ProtégéAI ACs vs. ECs.

Variable*	OAR					
	Brainstem	Chiasm	Eye L	Eye R	Larynx	Lens L
**DSC**	0.81 ± 0.04	0.30 ± 0.14	0.89 ± 0.03	0.89 ± 0.02	0.65 ± 0.09	0.55 ± 0.27
**MDs (mm)**	1.04 ± 0.29	1.33 ± 0.95	0.47 ± 0.20	0.44 ± 0.19	2.51 ± 0.97	0.38 ± 0.17
	Lens R	Mandible	Optic Nerve L	Optic Nerve R	Oral Cavity	Parotid L
**DSC**	0.54 ± 0.29	0.91 ± 0.03	0.53 ± 0.09	0.63 ± 0.13	0.82 ± 0.06	0.77 ± 0.13
**MDs (mm)**	0.43 ± 0.40	0.62 ± 0.32	0.54 ± 0.34	0.40 ± 0.22	2.04 ± 0.71	1.31 ± 0.74
	Parotid R	Spinal Cord	Submandibular L	Submandibular R	Esophagus	
**DSC**	0.79 ± 0.09	0.79 ± 0.05	0.68 ± 0.23	0.70 ± 0.28	0.75 ± 0.11	
**MDs (mm)**	1.44 ± 1.57	1.49 ± 4.55	2.41 ± 5.42	1.91 ± 3.36	0.76 ± 0.50	

*Mean ± Standard Deviation.

## 4 Discussion

Both ABAS and DLAS methods have shown promise in reducing variability and time required to establish contours (22–25). This study aimed to clinically validate two DLAS commercial software programs by comparing automatically-generated OAR contours with those created manually for a cohort of 40 HNC patients. DLAS contours were evaluated with two gold-standard geometric measures. Reasonable agreement was shown for the glandular OARs, eyes, brainstem, spinal cord, oral cavity, esophagus, and mandible across both autocontour programs. Moderate agreement was shown for the optic nerves and lenses, constrictor muscle, and larynx (ProtégéAI specifically). There was poor agreement for the larynx (AccuContour specifically) and chiasm. The results demonstrate that each DLAS package can adequately contour most HNC OARs efficiently in an independent cohort of patients.

We were able to provide results that closely resemble those reported in other DLAS studies in the head and neck region. There is a consensus on the efficiency of DLAS programs to contour the mandible and brainstem as reported by Brunenberg et al. (26) (DSC 0.95 and 0.87, respectively) validating another commercially-available DLAS program, DLCExpert™. The results for the glandular OARs and optic nerves in this study resemble those reported by Ibragimov and Xing (15) (DSC parotid gland 0.78, submandibular gland 0.73, optic nerve 0.65) using a convolutional neural network (CNN) approach. Willems et al. (27) reported pharyngeal constrictor muscle results (inferior, middle, and superior constrictor muscle average DSC 0.55) that closely resemble the overall constrictor muscle ACs generated in this study. Overall, structures with larger volumes appeared to be easier for the DLAS models to contour as given by the larger DSCs.

**Figure 2:**
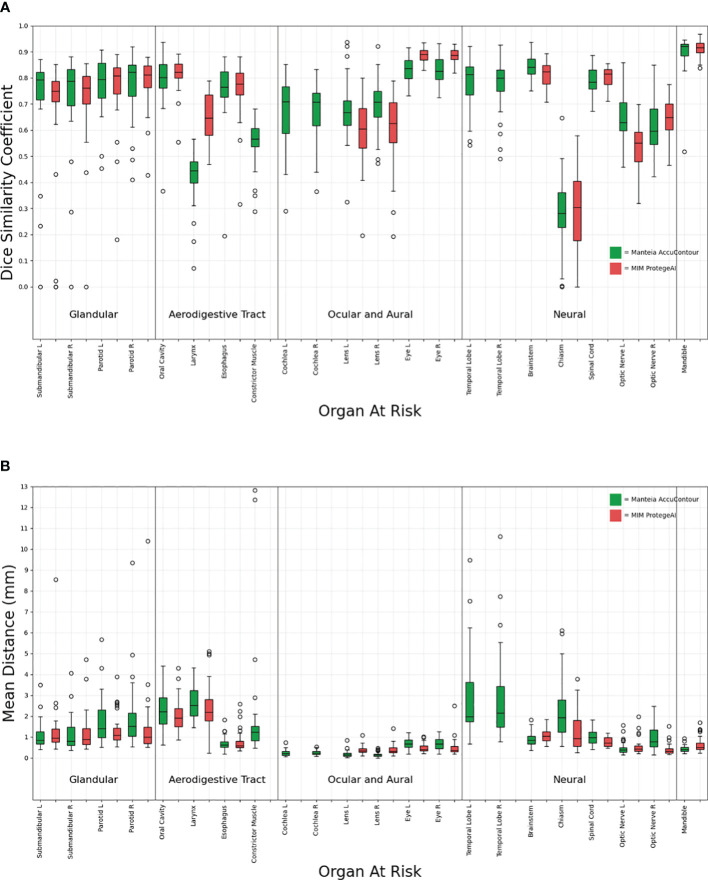
Comparison of Manteia AccuContour and MIM ProtègèAI for various OARs Sørensen-Dice similarity coefficients **(A)**, and Mean Distances **(B)**.

**Figure 3:**
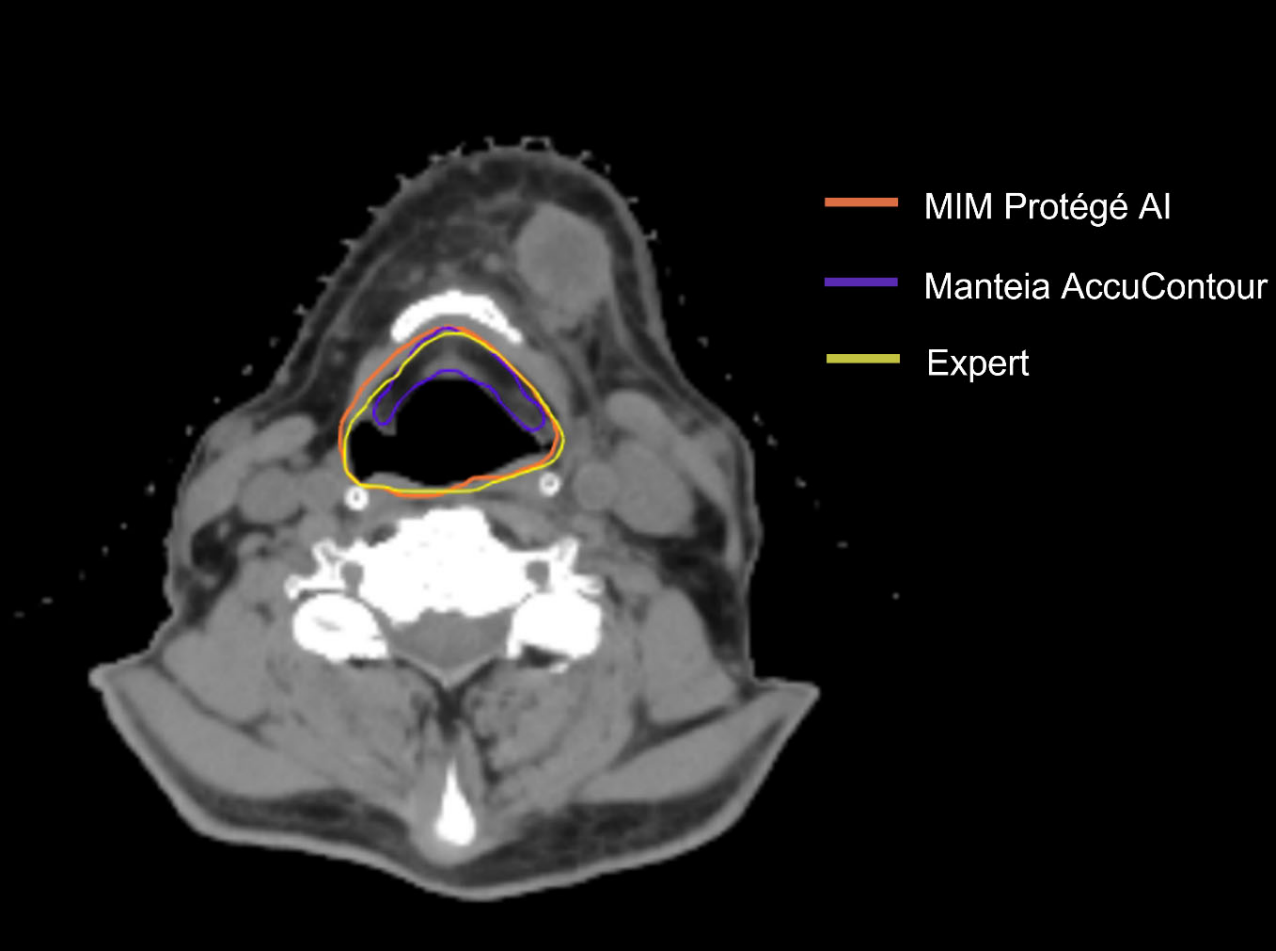
Comparison of AC and EC Larynx contour.

**Figure 2** shows that all the ACs generated by MIM ProtégéAI and Manteia AccuContour were comparable except for the larynx. The average DSC for the larynx contoured by AccuContour was noticeably lower than that of the larynx contoured using ProtégéAI. When investigated further, it was noted that the larynx AC within AccuContour was consistently omitting the airspace within the larynx when generating a contour (**Figure 3**). Our institution’s standard of practice for contouring the larynx is to include the entire structure as well as the airspace within. This apparent discrepancy led to less agreement between the EC and AC for the larynx using AccuContour, while ProtégéAI was able to contour in a similar fashion to our experts. Fortunately, Mantiea’s AccuLearning software allows for the creation of in-house models. Should we want to establish a DLAS model using our institution’s method of contouring the larynx, we would be able to do so in the future.

**Figure 4:**
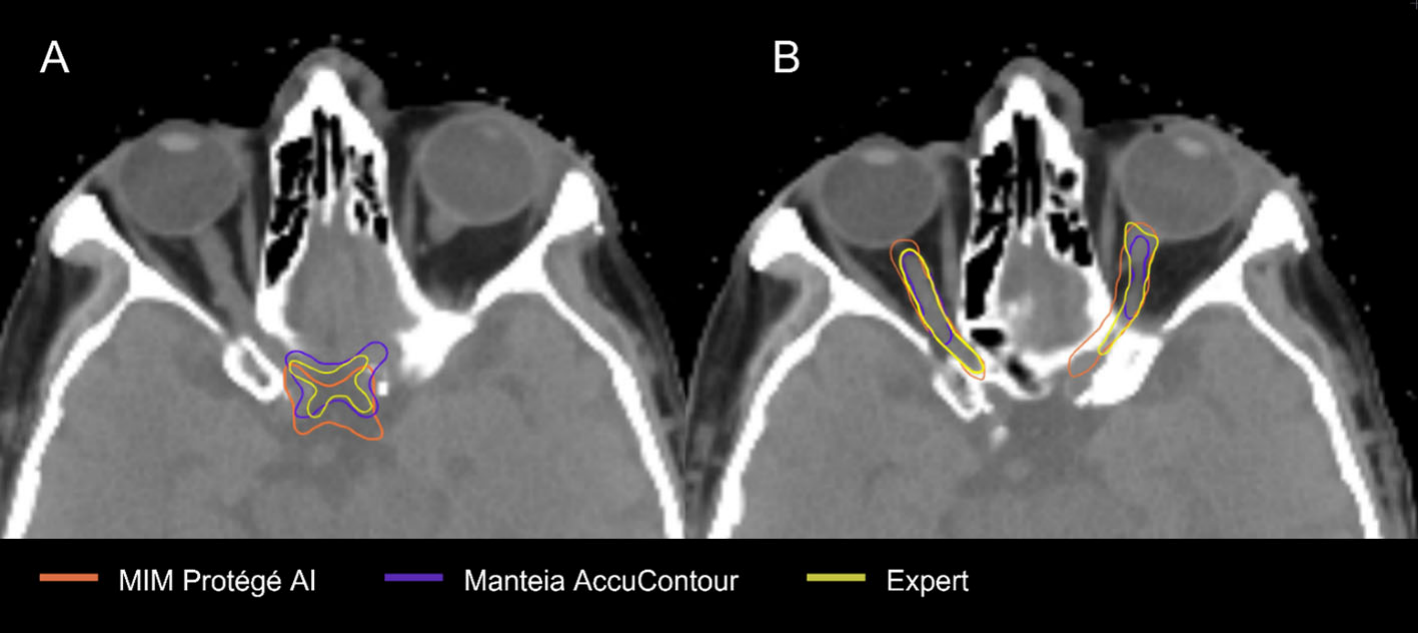
Comparison of EC and AC of Chiasm **(A)** and Optic Nerves **(B)** EC.

The OAR contours from each DLAS model which suffered the most were the chiasm and optic nerves (**Figure 4**). The low average DSCs of these structures do not appear unique to our study, as several other papers have reported low values for the chiasm and optic nerves (28–30). This can likely be attributed to small volumes of these structures as well as low contrast to the surrounding brain tissue in CT, making it difficult to accurately segment the structures as noted by a study from Ren et al (31). In this study, investigators were able to improve small structure DSCs (including the chiasm and optic nerves) using a specialized 3D CNN approach. In a 2018 study aiming to improve segmentation for small volume structures in the head and neck region, Tong et al. (28) were able to rescue the low DSC of the chiasm and optic nerves to an extent by employing a Shape Representation Model (SRM) to a Fully Convolutional Neural Network (FCNN), improving the average DSC for these small structures. Thus, further effort to adjust autocontouring solutions to accurately contour structures with small volumens appears advantageous.

Our study does have potential limitations. First, the generic HNC model within MIM ProtégéAI does not currently contain the temporal lobes, constrictor muscle, or cochlea. However, MIM’s research & development team has confirmed that these structures will be included in future updates and can be clinically validated at that time. We also only validated each model on 40 pCTs from one institution, thus limiting the power and generalizability of our results. Lastly, additional manual editing of ACs after creation was not performed, and only the first iteration of ACs generated was compared.

Thus, one consideration for future use is the practice of assessing ACs with additional manual editing where needed. This practice has proved useful in improving contour acceptability in previous studies. Willems et al. (27) recorded the time required to correct ACs as well as resulting DSCs and found that only an additional 15 minutes total were required to improve the ACs. This was also confirmed in a study by Teguh et al. (22), where the ACs were edited, improving similarity metrics while still requiring less time than manually creating contours from scratch. We did not routinely edit the contours generated by ProtégéAI or AccuContour, but future work could include this practice to assess time sparing and contour improvements.

While the auto-segmentation similarity metrics investigated in this study are commonly used, future research should incorporate advanced comparison metrics such as the surface DSC, as described by Vaassen et al. (32). A comprehensive overview of additional metrics is provided by Kiser et al. (33), who corroborate previous findings that surface DSC may better correlate with clinical applicability. Additionally, this study focused on the performance of commercially available auto-segmentation solutions; however, there is a growing trend of research teams developing open-source solutions, available on platforms like GitHub (34, 35). These open-source solutions often yield competitive results, rivalling commercial software and may be of particularly beneficial for research studies with limited funding or in clinics with constrained resources. Beyond the two commercial artificial intelligence based auto-segmentation software evaluated in this study, other options such Mirada, MVsision, Radformation, Raystation and TheraPanacea also provide contours of comparable quality for the organs at risk (OARs) in the head and neck region (36). The structures covered by each system are adequate for clinical application and can be customized for specific anatomical sites. The reported accuracy metrics of each system should be considered as one of the critical factors in the decision-making process. Additionally, institutions should evaluate the cost, service quality, and integration capability with existing clinical workflows when selecting an auto-segmentation solution.

## 5 Conclusion

Both commercially available DLAS programs were able to significantly reduce the time required to generate OAR contours, even though manual editing of ACs is likely needed prior to implementation into the clinic. The DSCs and MDs achieved were similar to those reported in other studies that evaluated various other DLAS solutions. Still, structures with small volumes are difficult to generate accurate ACs for and will require additional solutions to achieve sufficient contours.

## Data availability statement

The original contributions presented in the study are included in the article/supplementary material. Further inquiries can be directed to the corresponding author.

## Author contributions

CJ: Data curation, Formal analysis, Writing – original draft. RP: Data curation, Validation, Visualization, Writing – review & editing. CS: Supervision, Validation, Visualization, Writing – review & editing. BS: Data curation, Formal analysis, Visualization, Writing – review & editing. PT: Formal analysis, Validation, Writing – review & editing. LH: Data curation, Formal analysis, Validation, Writing – review & editing. FY: Data curation, Formal analysis, Writing – review & editing. CAp: Data curation, Formal analysis, Writing – review & editing. CAc: Data curation, Formal analysis, Writing – review & editing. HZ: Data curation, Formal analysis, Writing – review & editing. HL: Supervision, Writing – original draft, Writing – review & editing. SH: Conceptualization, Supervision, Writing – original draft, Writing – review & editing.
